# Fracture Tables

**Published:** 1853-10

**Authors:** 


### REVIEWS.

---

Art. VI.—*Fracture Tables.* By Frank H. Hamilton, A.M., M.D., Professor of Surgery in the University of Buffalo, and Surgeon to the Buffalo Hospital of the Sisters of Charity; with a Supplement, compiled from Dr. Hamilton's Notes, by John Boardman, A. B.; Comprising in all an Analysis of 461 Cases of Fractures. Buffalo: Printed by Jewett, Thomas & Co., 1853. 8vo., pp. 36.

This is the second edition of these instructive Tables, which are much enlarged by the addition of a Supplement. The facts which they embody are highly important in a practical point of view, and though the conclusion to which they seem to bring us in regard to the results of fractures is not one flattering to our professional pride, still it ought to be known. It is time we had determined how much and how little can be done by our art in the restoration of fractured bones. If the public have expected too much of the surgeon, and if the surgeon has been betrayed into promising too much, it is time that all parties were aware of the fact. Suits for malpractice, arising out of the treatment of broken limbs, occur almost every month in the year and every where in our country. Practitioners of experience and of unquestioned honesty are testifying constantly to having treated ten, twenty, or more, broken legs or thighs, without the occurrence of shortening, or any other deformity or accident. Do they testify to the truth, or have they deceived themselves? Is such perfection, in the management of such casualities, attainable? Statistics give an unfavorable answer. These "Tables" testify that perfect restoration after such accidents is rare. Practitioners deceive themselves; they do not measure the broken limbs after recovery, or perhaps lose sight of their patients before they are considered entirely well, and congratulate themselves on their success when a limping gait tells to some other community quite another story. It is not pleasant to believe this. We wish we could feel some assurance that surgical art had attained to something like perfection in this department, but we owe it to ourselves not to deceive ourselves. If we profess too much, we can expect nothing else but to be prosecuted by our patients when we fail to restore their limbs in their full integrity. When we hear a surgeon claiming to have compassed perfect success by a particular form of instruments, or by dispensing with all instruments, we confess we cannot avoid a suspicion that he is deceiving himself. His process loses its efficacy when tried by other hands. There is but one infallible test in all these cases — let the limbs be measured, and let us have the statistics.

Dr. Hamilton has rendered an important service to the profession by this analysis. We shall learn where we stand by such a procedure, and shall be made to feel the necessity of yet further improvement in this branch of our art. As the result of his researches on this subject, Dr. H. believes that in fifty cases of fracture of the lower extremities, forty, at least, will be found more or less bent or shortened.

In fourteen cases of fracture of the *Humerus*, the results were: in one, elbow stiff; in another, arm much bent at seat of fracture, inner half of arm and two fingers numb, power of arm much impaired; in a third, arm slightly bent, and not as strong as the other; in a fourth, external condyle displaced, and joint rather stiff; in a fifth, elbow anchylosed; in a sixth, muscles wasted, arm cannot be perfectly straightened, grating between the articular surfaces; in the seventh, the arm was bent slightly inward; and in two others, there were imperfections more or less serious; making nine out of fourteen.

Fractures of the Radius. Here, out of eight cases, there were three of imperfect recovery.

The Ulna afforded but one imperfect case in nine.

Radius and Ulna together, six in which there was defect, against seven in which the restoration was perfect.

Neck of the Femur, seven cases, and all imperfect. Every variety of apparatus was tried in these cases. In one the patient died before leaving her bed. In one, where no splint was used, the patient has not used the limb since the accident, and is bed ridden. In some there were prosecutions for malpractice.

Of the Shaft of the Femur, twenty-one cases are given, and all but four are marked "imperfect." The limbs were shortened, or bent, or there was anchylosis of the knee, or turning in of the foot, and in one case there was prosecution of the surgeon.

Two cases of fracture of the Patella are given, in both of which there was imperfection.

Fracture of the Leg, in twenty-one cases, afforded seventeen instances of imperfect recovery, and two prosecutions.

Clavicle. Twenty-three instances of fracture of this bone furnish fourteen imperfect cases.

Of fracture of the Humerus twenty-four cases are cited in the Supplement, and twelve are recorded as imperfect.

The Radius, fractured in nineteen instances, was imperfectly restored in eight. The Ulna eight in fourteen.

The Radius and Ulna together give but three cases of imperfect recovery in twenty, seventeen having resulted in perfect restoration of the limb.

The Femur, in forty-three instances, shows nine of perfect recovery.

The Leg was perfectly restored in eighteen out of fifty cases.

In the subjoined table there is an interesting summing up of the facts on this subject —

SUMMARY OF TABLES OF ALL THE CASES FROM THE NOTES OF DR. HAMILTON.

| Name of Bones. | No. of Fractures. | No. of Perfect. | No. of Imperfect. | Points of Fracture. | | | Character of Fracture. | | | Relative No. at Different Ages. | | | | | | | | | |
|---|---|---|---|---|---|---|---|---|---|---|---|---|---|---|---|---|---|---|---|
| | | | | Upper or outer $\frac{1}{3}$. | Middle. | Lower $\frac{1}{3}$. | Comp. and Commin. | Comp. | Simple. | To 15 years. | | 15 to 30 years. | | 30 to 45 years. | | 45 to 60 years. | | 60 to 82 years. | |
| | | | | | | | | | | No. | P. | No | P. | No. | P. | No | P. | No | P. |
| Ossa Nasi.... | 9 | 1 | 8 | .. | .. | .. | .. | 3 | 6 | 5 | 1 | 3 | .. | .. | .. | .. | .. | .. | .. |
| Septum Nasi. | 4 | 1 | 3 | .. | .. | .. | .. | .. | 4 | 3 | .. | 1 | .. | .. | .. | .. | .. | .. | .. |
| Sup. Maxilla. | 1 | 1 | .. | .. | .. | .. | .. | .. | 1 | .. | .. | .. | .. | 1 | 1 | .. | .. | .. | .. |
| Inf. Maxilla.. | 13 | 7 | 6 | .. | .. | .. | 6 | 3 | 1 | 2 | 2 | 7 | 4 | 3 | 1 | 1 | .. | .. | .. |
| Clavicle. .... | 41 | 15 | 26 | 32 | 9 | .. | .. | 4 | 37 | 10 | 8 | 9 | 3 | 14 | 2 | 7 | 2 | .. | .. |
| Scapula ..... | 3 | 1 | 2 | .. | .. | .. | .. | .. | 3 | .. | .. | 2 | .. | .. | .. | .. | .. | 1 | 1 |
| Humerus .... | 38 | 17 | 21 | 5 | 7 | 26 | 2 | 1 | 36 | 18 | 8 | 5 | 3 | 10 | 4 | 3 | 2 | 2 | .. |
| Radius ...... | 27 | 17 | 10 | 3 | 4 | 20 | .. | 2 | 25 | 12 | 9 | 7 | 5 | 6 | 2 | 2 | .. | .. | .. |
| Ulna ........ | 23 | 14 | 9 | 10 | 6 | 6 | .. | 2 | 20 | 10 | 8 | 8 | 5 | 4 | 1 | 1 | .. | .. | .. |
| Radius & Ulna | 34 | 25 | 9 | 2 | 11 | 20 | .. | 2 | 31 | 23 | 18 | 5 | 3 | 5 | 3 | .. | .. | 1 | 1 |
| Femur ...... | 73 | 14 | 59 | 23 | 38 | 7 | 3 | 1 | 67 | 23 | 10 | 10 | 3 | 19 | 1 | 5 | .. | 6 | .. |
| Patella ...... | 7 | 1 | 6 | .. | .. | .. | .. | .. | 7 | 1 | .. | 2 | 1 | 4 | .. | 1 | .. | .. | .. |
| Tibia........ | 19 | 14 | 5 | 3 | 10 | 6 | .. | 6 | 13 | 3 | 2 | 9 | 5 | 3 | 3 | 5 | 4 | .. | .. |
| Fibula .... .. | 16 | 8 | 8 | 1 | 3 | 12 | 1 | 4 | 11 | 2 | | 4 | 2 | 6 | 4 | 2 | 2 | .. | .. |
| Tibia & Fibula | 73 | 23 | 50 | 7 | 20 | 4 | 13 | 19 | 40 | 11 | 5 | 15 | 5 | 22 | 5 | 1 | .. | .. | .. |
| Carpal ...... | 1 | .. | 1 | .. | .. | .. | 1 | .. | .. | .. | .. | 1 | .. | .. | .. | .. | .. | .. | .. |
| Metacarpal .. | 3 | 1 | 2 | 1 | .. | 2 | .. | 1 | 2 | 1 | .. | 2 | 1 | .. | .. | .. | .. | .. | .. |
| Phalanges ... | 1 | .. | 1 | .. | .. | .. | .. | 1 | .. | 1 | .. | .. | .. | .. | .. | .. | .. | .. | .. |
| Ribs ........ | 4 | 2 | 2 | .. | .. | .. | .. | .. | 4 | .. | .. | 1 | .. | 3 | 2 | .. | .. | .. | .. |
| Vertebra .... | 3 | .. | .. | .. | .. | .. | .. | .. | 3 | 2 | .. | .. | .. | . | .. | 1 | .. | .. | .. |
| Pelvis....... | 2 | .. | 2 | .. | .. | .. | .. | .. | 1 | .. | .. | .. | .. | 2 | .. | .. | .. | .. | .. |
| Amount ; | 395 | 161 | 231 | 87 | 108 | 144 | 26 | 49 | 312 | 126 | 71 | 93 | 46 | 102 | 29 | 29 | 10 | 10 | 2 |

Dr. Hamilton has brought the profession under obligations to him by the publication of these Tables.

